# Trophic overlap between fish and riparian spiders: potential impacts of an invasive fish on terrestrial consumers

**DOI:** 10.1002/ece3.1893

**Published:** 2016-02-17

**Authors:** Michelle C. Jackson, Darragh J. Woodford, Terence A. Bellingan, Olaf L. F. Weyl, Michael J. Potgieter, Nick A. Rivers‐Moore, Bruce R. Ellender, Hermina E. Fourie, Christian T. Chimimba

**Affiliations:** ^1^ Department of Zoology and EntomologyCentre for Invasion BiologyUniversity of PretoriaPrivate Bag X 20Hatfield0028South Africa; ^2^ School of Animal, Plant and Environmental SciencesUniversity of the WitwatersrandJohannesburgSouth Africa; ^3^ South African Institute for Aquatic Biodiversity (SAIAB)Grahamstown6140South Africa; ^4^ Centre for Invasion BiologySAIABGrahamstown6140South Africa; ^5^ Department of Zoology and EntomologyRhodes UniversityGrahamstown6140South Africa; ^6^ Centre for Water Resources ResearchUniversity of KwaZulu‐NatalPrivate Bag X01Scottsville3209South Africa

**Keywords:** Competition, invasive species, niche overlap, nicheROVER, stable isotope, trophic subsidies

## Abstract

Studies on resource sharing and partitioning generally consider species that occur in the same habitat. However, subsidies between linked habitats, such as streams and riparian zones, create potential for competition between populations which never directly interact. Evidence suggests that the abundance of riparian consumers declines after fish invasion and a subsequent increase in resource sharing of emerging insects. However, diet overlap has not been investigated. Here, we examine the trophic niche of native fish, invasive fish, and native spiders in South Africa using stable isotope analysis. We compared spider abundance and diet at upstream fishless and downstream fish sites and quantified niche overlap with invasive and native fish. Spider abundance was consistently higher at upstream fishless sites compared with paired downstream fish sites, suggesting that the fish reduced aquatic resource availability to riparian consumers. Spiders incorporated more aquatic than terrestrial insects in their diet, with aquatic insects accounting for 45–90% of spider mass. In three of four invaded trout rivers, we found that the average proportion of aquatic resources in web‐building spider diet was higher at fishless sites compared to fish sites. The probability of web‐building and ground spiders overlapping into the trophic niche of invasive brown and rainbow trout was as high as 26 and 51%, respectively. In contrast, the probability of spiders overlapping into the trophic niche of native fish was always less than 5%. Our results suggest that spiders share resources with invasive fish. In contrast, spiders had a low probability of trophic overlap with native fish indicating that the traits of invaders may be important in determining their influence on ecosystem subsidies. We have added to the growing body of evidence that invaders can have cross‐ecosystem impacts and demonstrated that this can be due to niche overlap.

## Introduction

Both direct competition and indirect resource competition typically reduce species fitness, and therefore, functionally similar species often coexist by niche partitioning. This separation reduces resource sharing and hence promotes co‐occurrence. Ecological studies on resource competition and niche partitioning generally consider species that occur in the same spatial habitat, for instance partitioning between freshwater fish (Jackson and Britton 2014), ungulates (Stewart et al. 2003) and seabirds (Cherel et al. 2008). However, due to the considerable flow of energy between adjacent habitats, such as streams and riparian zones (Baxter et al. 2005) and marine and coastal systems (Spiller et al. 2010), there is potential for resource competition between populations which never actually interact directly. This is particularly true of invaded systems, where drastic changes in community structure and ecosystem functioning (which includes productivity and decomposition; for example, Jackson et al. 2014) have the potential to cascade across ecosystem boundaries (Baxter et al. 2004; Benjamin et al. 2011).

The exchange of organisms and energy across ecosystem boundaries has major implications for food web structure and ecosystem dynamics (Baxter et al. 2005; Marczak et al. 2007; Bartels et al. 2012); food webs in streams and riparian habitats can be shaped by aquatic–terrestrial links (Knight et al. 2005). Terrestrial invertebrates that fall into streams are consumed by fish, and there is a reciprocal flow of energy in the form of adult aquatic insects emerging into the riparian habitat (Knight et al. 2005; Burdon and Harding 2008; Muehlbauer et al. 2014). These insects, such as chironomid flies, are an important resource for riparian species, often contributing 25–100% to the diet of birds, bats, and spiders (Grey et al. 2004; Baxter et al. 2005). Before they emerge, the larvae of insects may be consumed by predatory aquatic invertebrates and fish, and therefore, changes in aquatic community structure can influence insect emergence and, subsequently, resource availability to riparian consumers (Baxter et al. 2004; O'Callaghan et al. 2013; Gergs et al. 2014).

Invasive species may alter the flow of energy across aquatic–terrestrial boundaries, instigating trophic cascades that propagate through the food web (e.g., Baxter et al. 2004; Benjamin et al. 2011; Hladyz et al. 2011). Invasive fish often consume the aquatic larvae of insects, reducing the numbers that emerge as adults into the riparian habitat (Epanchin et al. 2010). In turn, this can instigate declines in the abundance of native riparian consumers, such as frogs, birds, and spiders, by reducing the abundance of an important food resource (Baxter et al. 2004; Finlay and Vredenburg 2007; Epanchin et al. 2010; Benjamin et al. 2011). Despite this evidence illustrating declines in riparian consumer abundance as a result of fish invasions, no studies have examined the diet of these adjacent populations in invaded ecosystems to investigate dietary overlap and niche partitioning (but see Gergs et al. 2014). Subsequently, the mechanism causing the observed declines in riparian consumer abundance has only been speculated. Therefore, here we use stable isotope analyses to examine the trophic ecology of fish and riparian spiders at stream sites with invasive, native, and no fish.

**Figure 1:**
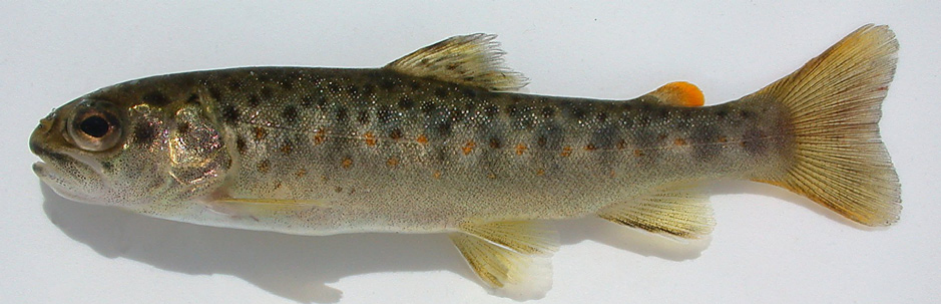
Juvenile non‐native brown trout (*Salmo trutta*) in South Africa.

The traits of spiders, which tend to be generalist predators, are likely to affect the extent of their dietary overlap with fish (Sanders et al. 2015). Similarly, the traits of fish will influence the availability of aquatic insects to riparian consumers (Benjamin et al. 2011). Consequently, we aimed to examine how (1) the hunting mode/feeding group of spiders influences their trophic overlap with fish and (2) whether the invasive or native status of the fish explained these differences. The invasive fish present in our study area were rainbow trout (*Oncorhynchus mykiss*) and brown trout (*Salmo trutta*; Fig. 1), both popular sport fish with invasive populations around the world, including South Africa, where they were introduced in the late 19th century (Ellender et al. 2014). Outside of their native range (i.e., North America and Asia/Europe), they often have adverse consequences for native fish, invertebrates, and ecosystem functioning (e.g., Ruzycki et al. 2003; Townsend 2003). In South Africa, studies have shown that introduced trout can alter the distribution, diversity, and abundance of native fish, invertebrates, and amphibians (Karssing et al. 2012; Rivers‐Moore et al. 2013; Shelton et al. 2015); however, as far as we are aware, no studies in Africa have quantified their cross‐ecosystem cascading impacts. We hypothesized that (1) spiders would have a higher probability of overlapping into the niche region of invasive fish when compared to native fish because the invaders are generalist predators with feeding traits not exhibited by native species; and (2) spider diet would shift toward terrestrial resources in the presence of fish due to a decline in emerging aquatic insect abundance.

## Materials and Methods

### Study sites

We studied tributaries of the Keiskamma (Eastern Cape Province), the Thukela, and the uMgeni (KwaZulu‐Natal Province) Rivers in South Africa. The surrounding area of all of the sites is characterized by indigenous forest and grassland. We sampled 12 sites across six streams in November 2013 (Keiskamma streams: Cata, Mnyameni and Gwiligwili) and April 2014 (Thukela and uMgeni streams; Sterkspruit, Mooi and Lotheni). Five of the streams have been invaded by trout to different degrees and the sixth (Gwiligwili) was used as an un‐invaded reference site. Within each stream, we sampled above and below a fish barrier (waterfalls >2 m), and therefore, our sites represented upstream fishless and downstream fish sites. Stream characteristics were standardized across the study scale as far as possible to isolate any impacts of trout invasion (see Table S1). All 12 sites were first‐ or second‐order streams, clear, 2–6 m wide with boulder and cobble substrates. We also measured stream pH, dissolved oxygen, and temperature at the time of sampling (Table S1). We estimated the percentage canopy cover and pool: riffle: cascade ratios, and local weather stations were used to obtain data on the average rainfall during the month of sampling (Table S1). At each of the sites, a 30‐m stream reach and the associated riparian area extending 1 m back from the water's edge was used for the sampling regime.

### Community structure

The fish community was sampled by backpack electrofishing. All fish were identified to species, measured, and counted. Muscle samples were taken from under the dorsal fin for subsequent stable isotope analysis. To determine the input of aquatic insects to the riparian habitat, four Surber samples were taken at riffles in each site to calculate the abundance of aquatic invertebrate larvae which have a terrestrial adult stage. The samples were preserved in 70% ethanol before identification to the family level. Additional invertebrates were collected using pond nets and frozen for later stable isotope analysis.

Two 10 × 1 m transects on either side of the stream at each site were searched for all ground spiders and web‐building spiders found to a maximum height of 2 m. The spiders were collected by hand and frozen for later identification to the family level before preparation for stable isotope analysis. Representative terrestrial insects which do not have an aquatic stage were also collected using a sweep net for subsequent stable isotope analysis. We used analysis of variance (ANOVA) to test for statistically significant differences in larval insect and spider abundance between upstream fishless and downstream fish sites in each stream.

### Stable isotope analyses

Carbon and nitrogen stable isotope analysis is a valuable tool to infer trophic links and characterize food web structure because the isotope signature of the body tissue of a consumer will reflect that of what it has eaten (essentially, “you are what you eat”). After being allowed to gut‐clear, all invertebrate samples were analyzed whole. All samples were oven dried at 60°C to constant weight, ground to a homogeneous powder and either 0.6 mg or 1 mg weighed into ultra‐clean tin cups for animal and plant material, respectively. Carbon and nitrogen stable isotope analysis was then conducted using a mass spectrometer coupled to an elemental analysis at the Mammal Research Institute (MRI), University of Pretoria, South Africa. The data outputs were in the format of delta (*δ*) isotope ratios expressed per mille (‰).

We compared spider diet within rivers at upstream fishless and downstream fish sites using isotope mixing models. To account for spatial variability in isotopic baselines between the sites at each river, we only used rivers where (1) terrestrial and aquatic insect resources had significantly different isotope signatures at each site and (2) downstream and upstream resources were statistically indistinguishable (tested using ANOVA; Table S2). Four rivers met this criteria (Cata, Mooi, Lotheni, and Mnyameni) allowing us to compare spider diet in the presence and absence of fish using an isotope mixing model. We used the Bayesian mixing model SIAR in the R computing program (Parnell et al. 2010; R Core Team 2015) to calculate the relative contribution of pooled aquatic and terrestrial insect resources to the average diet of spider populations at the eight sites. Resources were pooled for each habitat (aquatic and terrestrial) at each site because they overlapped in isotopic space. We used trophic fractionation values for invertebrate terrestrial consumers of 0.5 ± 0.19‰ for *δ* ^13^C and 2.3 ±  0.24‰ for *δ* ^15^N based on a review by McCutchan et al. (2003).

For each downstream site, we also calculated the isotopic niche region (*N*_R_) that each group occupied using the nicheROVER package in the R computing program (R Core Team 2015; Swanson et al. 2015). The groups were classified at the species level for fish and as functional feeding groups for spiders (web builders or ground spiders). Spider size (measured as total dry weight) had no influence on either *δ* ^15^N or *δ* ^13^C across all sites (*R* ^2^ = 0.00 for both, *n* = 225) and therefore was not considered further in our analyses. Each group was analyzed separately across the six sites to account for any temporal and spatial variation in isotopic baselines. *N*_R_ is defined as a 95% probability niche region in isotopic space. To account for uncertainty and sample size variations, for each group, 10 random elliptical projections of *N*_R_ were drawn using a Bayesian framework (Swanson et al. 2015). We then calculated overlap as the probability that individual web‐building and ground spiders are found in the *N*_R_ of each fish species at downstream sites in each river (Swanson et al. 2015).

## Results

### Status of streams

Stream characteristics were similar between paired upstream and downstream sites in each stream (Table S1), and therefore, any differences can be attributed to either the presence or absence of fish. Four of the sampled streams contained brown trout (Cata, Lotheni, Mooi, and Sterkspruit) and one had rainbow trout (Mnyameni; Table S1). Native Natal mountain catfish (*Amphilius natalensis*) were also present at three sites (Lotheni, Mooi, and Sterkspruit; Table S1). The chubbyhead barb (*Barbus anoplus*) and Border barb (*Barbus trevelyani*) were the only two fish species recorded in the un‐invaded reference stream (Gwiligwili; Table S1). Mountain catfish in the Lotheni and Mooi rivers were not present in sufficient numbers for isotope analysis. Electrofishing confirmed that all upstream sites were fishless.

### Community structure

**Figure 2:**
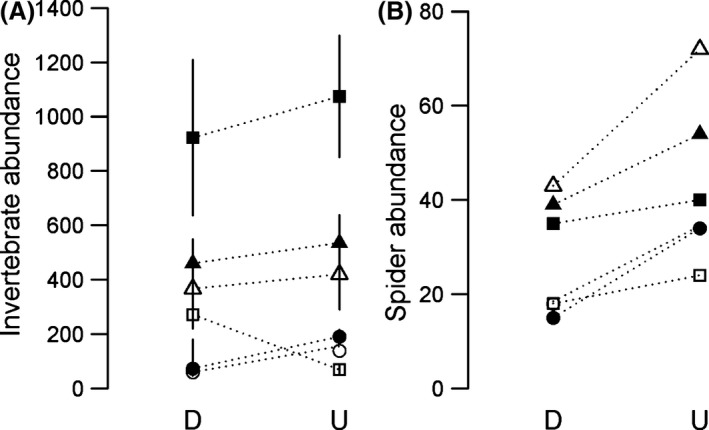
The abundance of aquatic larvae (A; mean ± standard error; *n *=* *4 or 5; per 1 m^2^) and spiders (B; total per 20 m^2^) at paired downstream (D) and upstream (U) sites in each river. Where open circles = Cata, closed circles = Mnyameni, open triangles = Mooi, closed triangles = Lotheni, closed squares = Sterkspruit and open squares = Gwiligwili. Dotted lines join paired upstream and downstream sites.

The abundance of emerging insect larvae did not vary significantly between paired upstream and downstream sites except in the Mnyameni River (ANOVA: *F*_1,6_ = 14.36, *P* = 0.009) where the abundance of insect larvae was lower at the downstream site invaded by rainbow trout. Regardless, the abundance of insect larvae was consistently higher at all fishless upstream sites except in the un‐invaded Gwiligwili River (Fig. 2A) despite the fact this river had the highest fish densities at the downstream site (Table S1). Spider abundance varied from 15 to 72 per 10 m^2^ and was significantly higher at all upstream sites (*F*_1,5_ = 17.13, *P* = 0.01; Fig. 2B). The most common web‐building spiders were of the families Tetragnathidae and Araneidae, both orb‐weaving families. The most common ground spiders were from the families Lycosidae and Pisauridae.

### Diet analysis

Our Bayesian mixing models revealed that aquatic insects are important in the diet of riparian spiders, contributing more than that of terrestrial insects in 14 of 16 populations (Table 1). The contribution of aquatic resources to web‐building spider population diet increased at upstream fishless sites in three of the four rivers analyzed (Table 1). In contrast, the contribution of aquatic resources to ground spider diet decreased at all four upstream fishless sites (Table 1).

**Table 1:** Bayesian mixing model output (mean [95% credible intervals]) of ground and web‐building spider population diet from four rivers invaded by trout showing the estimated proportion of aquatic resources at upstream fishless (up) and downstream invaded (down) sites

River	Site	Ground spiders		Web spiders	
		Proportion	*n*	Proportion	*n*
Cata	Down	84.9 (54.6–1.0)	9	90.0 (73.9–1.0)	9
	Up	45.4 (3.9–80.4)	3	66.4 (55.7–76.7)	31
Lotheni	Down	63.9 (40.7–89)	7	59.1 (30.8–93.4)	6
	Up	57.8 (38.8–77.2)	5	68.7 (52.6–83.7)	9
Mnyameni	Down	88.1 (73.7–1.0)	10	79.5 (57.6–1.0)	6
	Up	45.6 (9.4–77.7)	4	85.9 (74.5–97.5)	30
Mooi	Down	72.3 (46.0–98.7)	3	67.4 (40.9–96.7)	13
	Up	56.9 (28.0–88.0)	4	75.7 (66.2–84.7)	20

**Figure 3:**
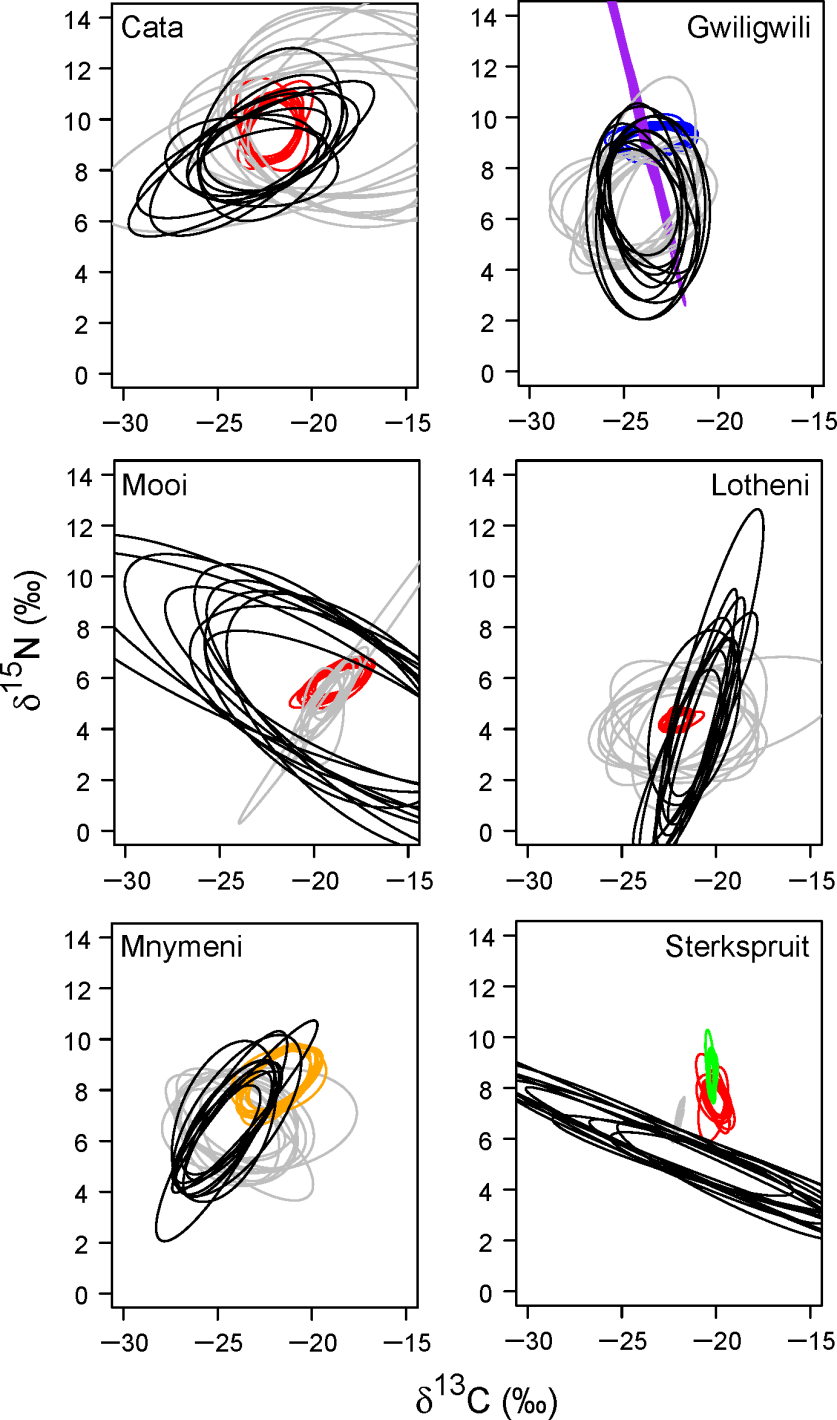
Ten random elliptical projections of trophic niche region (*N*_R_) for each group at the downstream sites in each stream. The groups displayed are brown trout (red), rainbow trout (orange), border barb (blue), chubby head barb (purple) mountain catfish (green), web‐building spiders (black) and ground spiders (gray).

The probability of individual spiders occurring in the *N*_R_ of fish varied across rivers and fish species (Table 2; Fig. 3). In general, higher probabilities of overlap occurred with invasive trout, reaching up to 51% in the Mooi River (Table 2). In contrast, the probability of overlap with native fish varied between 0 and 5% (Table 2). The Sterkspruit River was an exception, with no overlap projected between spiders and either native or invasive fish (Table 2; Fig. 3). The functional feeding group of spiders (web and ground spiders) did not appear to influence the probability of trophic overlap with fish (Table 2; Fig. 3).

**Table 2:** The probability (posterior means and 95% credible intervals) of individual riparian spiders from each functional group occurring in the niche region (*N*_R_) of adjacent fish species in each stream

Stream	FFG	Probability of overlap (%)				
		Invasive brown trout	Invasive rainbow trout	Native border barb	Native chubbyhead barb	Native mountain catfish
Cata	Ground	11.63 (4–18)	–	–	–	–
Cata	Web	25.5 (15–39)	–	–	–	–
Gwiligwili	Ground	–	–	1.80 (0–8)	1.84 (0–6)	–
Gwiligwili	Web	–	–	5.06 (0–15)	2.24 (0–7)	–
Mooi	Ground	50.55 (11–92)	–	–	–	–
Mooi	Web	8.27 (4–16)	–	–	–	–
Lotheni	Ground	8.31 (3–19)	–	–	–	–
Lotheni	Web	2.13 (0–8)	–	–	–	–
Mnyameni	Ground	–	10.78 (1–30)	–	–	–
Mnyameni	Web	–	22.01 (4–50)	–	–	–
Sterkspruit	Ground	0 (0–0)	–	–	–	0 (0–0)
Sterkspruit	Web	0.01 (0–1)	–	–	–	1 (0–0)

## Discussion

The cascading negative impacts of invasive fish on riparian consumers is well‐documented (Baxter et al. 2004; Epanchin et al. 2010; Benjamin et al. 2011), but the trophic interactions which mediate these impacts have only been speculated upon. Here, we have shown that native riparian spiders have a higher probability of sharing trophic niche space with invasive trout when compared to native fish. This suggests that invasive trout are more likely to reduce resource availability to, and compete with, riparian spiders than some native fish, potentially explaining the negative effects of trout on riparian consumers elsewhere.

The success of invasive species is often attributed to their plasticity in behavior and diet choice (e.g., Sol et al. 2002; Caut et al. 2008). Invasive fishes often have higher feeding rates (Alexander et al. 2014) and wider niche breadths (Carman et al. 2006; Jackson and Britton 2013; Hill et al. 2015) than native species which could explain their higher degree of niche overlap with spiders when compared to native fish. However, in streams where invasive trout are functionally redundant due to the presence of a functionally similar native fish, their impact on riparian consumers is likely to be undetectable. The native fish present at our sites are functionally different from the invaders, being primarily benthic insectivores or omnivorous (Gaigher 1975), whereas the trout are generalist predators that specialize in drift‐foraging (Elliott 1973; Bachman 1984). This suggests that invasive trout will have a greater impact on riparian consumers at sites which previously had no fish (Epanchin et al. 2010) or sites where the native fish community does not fully exploit emerging insects accessible in the drift as a resource. The higher probability of spider niche overlap with invasive fish compared to native fish may thus reflect a higher propensity of trout to consume insects as they emerge.

In a similar stable isotope study, Gergs et al. (2014) found that the invasive shrimp, *Dikerogammarus villosus*, reduced aquatic resource availability to riparian spiders resulting in a diet shift toward terrestrial insects. At sites with low invasion densities, aquatic insects contributed 60% to the diet of web‐building spiders (Gergs et al. 2014), a similar finding to our study (66–85%) and a study in New Zealand (58%; Collier et al. 2002). At sites with high invasion densities, Gergs et al. (2014) estimated that the contribution of aquatic resources was reduced to 10%. We found that the contribution of aquatic resources to spider diet also decreased slightly at invaded sites, but only in web‐building spiders. Moreover, a decline in total spider abundance at downstream sites suggests that trout limited the availability of insect prey to riparian spiders. However, counterintuitively we found that the importance of aquatic resources in ground spider diet actually increased at downstream sites with fish in all four rivers despite the importance of aquatic resources to ground spider diet. Past studies have also found that ground spiders obtain a significant amount of their body mass from consuming aquatic resources (e.g., Collier et al. 2002; Paetzold et al. 2005) and so the reason for this is unclear. One possible explanation is that the reduction in spider abundances in the presence of fish may reduce competition between spiders for aquatic resources, and therefore permit the remaining spiders to consume a higher percentage of aquatic material, despite it being less available.

The comparison of fishless and invaded sites makes it difficult to determine whether the impact of trout in this study was a result of their invasive status or simply a “fish” impact, although the impacts of native fish on prey and spider abundance on the Gwiligwili appeared small relative to the trout‐invaded streams. Nonetheless, past studies have shown a difference in the impacts of invasive and native fish (Baxter et al. 2004; Benjamin et al. 2011) and our aim here was to investigate the trophic ecology of spiders to understand why invasive fish often have adverse impacts on native consumers while native fish do not.

Our findings suggest that invasive trout can have subtle, but nonetheless detectable impacts on terrestrial food webs because successful invasions represent the introduction of new competitors to terrestrial riparian predators. Trophic interactions and competition for resources are important in structuring communities but the cascading impacts of these processes across ecosystem boundaries are rarely considered. Here, we have demonstrated that, dietary overlap and resource competition across the aquatic–terrestrial ecotone should thus be considered as a likely driver of the cross‐ecosystem impacts of invasive fishes.

## Conflict of Interest

None declared.

## Data Accessibility

All stable isotope data are available in the supplementary material.

## Supporting information


**Table S1.** Characteristics of the 12 sites during the month of sampling.
**Table S2.** Invertebrate resource isotope values and results of ANOVA's testing for differences between resources and sites.
**Table S3.** Carbon and nitrogen stable isotope values of fish and spiders used in the study.
