# Supplementary material for: Trophic overlap between fish and riparian spiders: potential impacts of an invasive fish on terrestrial consumers
Source: Ecol Evol. 2016 Feb 17;6(6):1745–52. doi: 10.1002/ece3.1893 (PMC4801975; doi:10.1002/ece3.1893)
Supplement: Supplementary file 1 — Table S1. Characteristics of the 12 sites during the month of sampling. Table S2. Invertebrate resource isotope values and results of ANOVA's testing for differences between resources and sites. Table S3. Carbon and nitrogen stable isotope values of fish and spiders used in the study. [file ECE3-6-1745-s001.docx]

**Supporting Information: Trophic overlap between fish and riparian spiders: potential impacts of an invasive fish on terrestrial consumers**

Jackson et al., 2015; mjackson@zoology.up.ac.za

**Table S1:** Characteristics of the 12 sites during the month of sampling.

| **River** | **Site** | **Fish present** | **Trout abundance (per 100m^2^)** | **Native fish abundance (per 100m^2^)** | **Altitude** | **Total precipitation** | **Average water temperature** | **Average pH** | **Average DO** | **Average chlorophyll-a concentration** | **Canopy cover** | **Riffle:Pool:Cascade** |
| --- | --- | --- | --- | --- | --- | --- | --- | --- | --- | --- | --- | --- |
| Cata | Down | Brown trout | 5.79 | 0 | 889 | 113 | 11 | 7.0 | 12.6 | 0.3 | 70 | 57:43:00 |
| Cata | Up | --------- | 0 | 0 | 980 | 113 | 12.1 | 6.8 | 12.9 | 0.6 | 90 | 53:47:00 |
| Gwiligwili | Down | Chubby head barb, Border barb | 0 | 49.0 | 700 | 113 | 12.8 | 7.0 | 12.2 | 0.4 | 70 | 20:80:00 |
| Gwiligwili | Up | --------- | 0 | 0 | 776 | 113 | 11.9 | 7.0 | 12.3 | 0.5 | 76 | 13:87:00 |
| Lotheni | Down | Brown trout, Mountain catfish | 7.25 | 0.91 | 1593 | 17 | 10 | 8.0 | 11.9 | 1.4 | 10 | 45:45:10 |
| Lotheni | Up | --------- | 0 | 0 | 1636 | 17 | 10 | 8.0 | 11.2 | 0.4 | 10 | 95:05:00 |
| Mnyameni | Down | Rainbow trout | 3.06 | 0 | 944 | 113 | 12.3 | 7.5 | 12.1 | 2.2 | 80 | 40:60:00 |
| Mnyameni | Up | --------- | 0 | 0 | 1013 | 113 | 11.7 | 6.7 | 12.4 | 1.0 | 87 | 73:27:00 |
| Mooi | Down | Brown trout, Mountain catfish | 7.66 | 0.51 | 1784 | 17 | 9.5 | 7.8 | 11.2 | 2.2 | 10 | 80:20:00 |
| Mooi | Up | --------- | 0 | 0 | 1822 | 17 | 9.3 | 7.9 | 10.4 | 1.8 | 10 | 40:40:20 |
| Sterkspruit | Down | Brown trout, Mountain catfish | 3.25 | 7.3 | 1257 | 38 | 10.2 | 7.9 | 11.3 | 1.4 | 15 | 85:10:05 |
| Sterkspruit | Up | --------- | 0 | 0 | 1383 | 38 | 10.1 | 8.0 | 10.3 | 0.5 | 25 | 65:30:05 |

**Table S2:** Invertebrate resource isotope values (mean and standard deviation) and results of ANOVA’s testing for differences between upstream and downstream resources within each river (n = 6) and riparian habitat (n = 6), and for differences in aquatic and terrestrial resources at each site (n = 12). Statistically similar groups are indicated by the same colour text or background. Note that at four rivers (Cata, Mooi, Lotheni and Mnyameni) aquatic and terrestrial resources are significantly different in terms of δ^13^C and/or δ^15^N while resources at upstream and downstream sites within the rivers have similar δ^13^C and δ^15^N values. As these rivers have consistent baselines they were used to compare spider diet in the presence and absence of fish.

| **River** | **Isotope** | **Upstream aquatic** | **Upstream terrestrial** | **Downstream aquatic** | **Downstream terrestrial** | **df** | **P** |
| --- | --- | --- | --- | --- | --- | --- | --- |
| Cata | δ^15^N | 5.13 (1.27) | 5.09 (1.36) | 5.98 (1.35) | 4.00 (0.45) | 3,62 | 0.025 |
|  | δ^13^C | -24.29 (1.22) | -27.58 (2.65) | -24.70 (0.92) | -27.11 (1.46) | 3,62 | <0.001 |
| Gwiligwili | δ^15^N | 4.91 (1.31) | 4.00 (1.32) | 6.42 (0.96) | 4.50 (1.51) | 3,36 | <0.001 |
|  | δ^13^C | -28.03 (1.87) | -26.87 (0.68) | -26.15 (0.99) | -25.10 (2.86) | 3,36 | 0.003 |
| Mooi | δ^15^N | 2.50 (1.36) | 1.81 (3.16) | 2.47 (0.98) | 4.18 (1.88) | 3,26 | 0.25 |
|  | δ^13^C | -23.52 (1.61) | -17.09 (4.50) | -20.81 (1.47) | -15.44 (2.68) | 3,26 | <0.001 |
| Lotheni | δ^15^N | 0.83 (1.05) | 3.14 (0.28) | 1.09 (1.54) | 4.25 (2.51) | 3,27 | 0.001 |
|  | δ^13^C | -25.98 (1.46) | -19.03 (5.91) | -24.45 (1.86) | -22.01 (4.31) | 3,27 | 0.002 |
| Mnyameni | δ^15^N | 4.96 (1.37) | 4.47 (2.08) | 4.75 (0.95) | 2.31 (1.30) | 3,61 | 0.005 |
|  | δ^13^C | -24.87 (1.10) | -27.48 (2.03) | -24.34 (1.40) | -28.22 (2.61) | 3,61 | <0.001 |
| Sterkspruit | δ^15^N | 1.47 (0.59) | 2.03 (4.26) | 3.99 (1.28) | 3.98 (2.71) | 3,38 | <0.001 |
|  | δ^13^C | -27.53 (2.13) | -24.07 (4.43) | -24.49 (2.00) | -15.19 (5.12) | 3,38 | <0.001 |

**Table S3:** Carbon and nitrogen isotope values of the individual fish and spiders collected for the study.

| **River** | **Site** | **Group** | **Nitrogen** | **Carbon** |
| --- | --- | --- | --- | --- |
| Cata | Downstream | Brown trout | 8.73 | -22.27 |
| Cata | Downstream | Brown trout | 8.79 | -22.46 |
| Cata | Downstream | Brown trout | 8.70 | -23.07 |
| Cata | Downstream | Brown trout | 8.56 | -21.50 |
| Cata | Downstream | Brown trout | 8.88 | -21.35 |
| Cata | Downstream | Brown trout | 9.31 | -22.87 |
| Cata | Downstream | Brown trout | 8.71 | -21.26 |
| Cata | Downstream | Brown trout | 9.52 | -21.35 |
| Cata | Downstream | Brown trout | 10.52 | -22.08 |
| Cata | Downstream | Brown trout | 9.97 | -22.42 |
| Cata | Downstream | Brown trout | 9.84 | -22.23 |
| Cata | Downstream | Brown trout | 9.30 | -22.62 |
| Cata | Downstream | Brown trout | 10.03 | -21.60 |
| Cata | Downstream | Brown trout | 10.07 | -22.80 |
| Cata | Downstream | Brown trout | 9.86 | -21.40 |
| Cata | Downstream | Brown trout | 9.67 | -21.78 |
| Cata | Downstream | Brown trout | 9.75 | -22.20 |
| Cata | Downstream | Brown trout | 9.12 | -22.64 |
| Cata | Downstream | Brown trout | 9.64 | -22.04 |
| Cata | Downstream | Brown trout | 9.63 | -22.78 |
| Cata | Downstream | Brown trout | 9.64 | -22.62 |
| Cata | Downstream | Brown trout | 9.81 | -22.17 |
| Cata | Downstream | Brown trout | 9.57 | -22.75 |
| Cata | Downstream | Brown trout | 10.39 | -22.41 |
| Cata | Downstream | Brown trout | 9.76 | -22.35 |
| Cata | Downstream | Brown trout | 9.69 | -21.81 |
| Cata | Downstream | Brown trout | 9.68 | -21.84 |
| Cata | Downstream | Brown trout | 9.43 | -22.04 |
| Cata | Downstream | Brown trout | 10.54 | -21.56 |
| Cata | Downstream | Brown trout | 10.59 | -20.93 |
| Cata | Downstream | Brown trout | 10.21 | -21.26 |
| Cata | Downstream | Ground spider | 8.74 | -20.26 |
| Cata | Downstream | Ground spider | 8.45 | -16.96 |
| Cata | Downstream | Ground spider | 8.87 | -13.90 |
| Cata | Downstream | Ground spider | 12.39 | -19.41 |
| Cata | Downstream | Ground spider | 10.42 | -13.99 |
| Cata | Downstream | Ground spider | 9.43 | -15.82 |
| Cata | Downstream | Ground spider | 8.46 | -23.39 |
| Cata | Downstream | Ground spider | 9.18 | -21.82 |
| Cata | Downstream | Ground spider | 12.29 | -19.44 |
| Cata | Downstream | Web Spider | 8.74 | -24.51 |
| Cata | Downstream | Web Spider | 7.38 | -24.31 |
| Cata | Downstream | Web Spider | 8.13 | -23.46 |
| Cata | Downstream | Web Spider | 7.43 | -24.54 |
| Cata | Downstream | Web Spider | 9.52 | -21.23 |
| Cata | Downstream | Web Spider | 9.54 | -18.27 |
| Cata | Downstream | Web Spider | 8.43 | -20.15 |
| Cata | Downstream | Web Spider | 10.58 | -21.94 |
| Cata | Downstream | Web Spider | 8.45 | -22.95 |
| Cata | Upstream | Ground spider | 5.71 | -25.20 |
| Cata | Upstream | Ground spider | 5.66 | -26.00 |
| Cata | Upstream | Ground spider | 5.67 | -27.41 |
| Cata | Upstream | Web Spider | 6.74 | -25.40 |
| Cata | Upstream | Web Spider | 9.21 | -24.98 |
| Cata | Upstream | Web Spider | 7.83 | -24.45 |
| Cata | Upstream | Web Spider | 7.14 | -25.56 |
| Cata | Upstream | Web Spider | 6.65 | -25.10 |
| Cata | Upstream | Web Spider | 7.17 | -24.82 |
| Cata | Upstream | Web Spider | 5.34 | -25.82 |
| Cata | Upstream | Web Spider | 6.72 | -25.22 |
| Cata | Upstream | Web Spider | 6.05 | -25.41 |
| Cata | Upstream | Web Spider | 5.99 | -24.52 |
| Cata | Upstream | Web Spider | 6.71 | -24.31 |
| Cata | Upstream | Web Spider | 6.76 | -24.87 |
| Cata | Upstream | Web Spider | 6.17 | -25.14 |
| Cata | Upstream | Web Spider | 4.78 | -25.87 |
| Cata | Upstream | Web Spider | 6.63 | -24.04 |
| Cata | Upstream | Web Spider | 7.38 | -24.89 |
| Cata | Upstream | Web Spider | 6.32 | -24.52 |
| Cata | Upstream | Web Spider | 6.09 | -25.96 |
| Cata | Upstream | Web Spider | 4.27 | -24.87 |
| Cata | Upstream | Web Spider | 5.83 | -24.81 |
| Cata | Upstream | Web Spider | 7.29 | -24.53 |
| Cata | Upstream | Web Spider | 6.22 | -25.66 |
| Cata | Upstream | Web Spider | 6.57 | -24.79 |
| Cata | Upstream | Web Spider | 6.37 | -24.74 |
| Cata | Upstream | Web Spider | 4.69 | -25.67 |
| Cata | Upstream | Web Spider | 5.76 | -24.62 |
| Cata | Upstream | Web Spider | 5.27 | -25.78 |
| Cata | Upstream | Web Spider | 5.97 | -25.94 |
| Cata | Upstream | Web Spider | 7.32 | -25.13 |
| Cata | Upstream | Web Spider | 6.24 | -24.86 |
| Cata | Upstream | Web Spider | 4.99 | -26.83 |
| Gwiligwili | Downstream | Border barb | 9.06 | -24.07 |
| Gwiligwili | Downstream | Border barb | 9.41 | -22.53 |
| Gwiligwili | Downstream | Border barb | 8.77 | -24.82 |
| Gwiligwili | Downstream | Border barb | 8.53 | -23.88 |
| Gwiligwili | Downstream | Border barb | 8.94 | -24.33 |
| Gwiligwili | Downstream | Border barb | 9.47 | -23.46 |
| Gwiligwili | Downstream | Border barb | 8.78 | -23.99 |
| Gwiligwili | Downstream | Border barb | 9.35 | -23.16 |
| Gwiligwili | Downstream | Border barb | 9.60 | -23.45 |
| Gwiligwili | Downstream | Border barb | 9.55 | -22.93 |
| Gwiligwili | Downstream | Border barb | 9.17 | -22.82 |
| Gwiligwili | Downstream | Border barb | 9.02 | -22.62 |
| Gwiligwili | Downstream | Border barb | 9.15 | -22.12 |
| Gwiligwili | Downstream | Border barb | 8.94 | -22.54 |
| Gwiligwili | Downstream | Border barb | 8.83 | -23.21 |
| Gwiligwili | Downstream | Border barb | 8.96 | -22.35 |
| Gwiligwili | Downstream | Border barb | 8.98 | -22.96 |
| Gwiligwili | Downstream | Border barb | 8.95 | -24.76 |
| Gwiligwili | Downstream | Border barb | 9.27 | -22.04 |
| Gwiligwili | Downstream | Border barb | 9.17 | -24.03 |
| Gwiligwili | Downstream | Border barb | 9.01 | -24.75 |
| Gwiligwili | Downstream | Border barb | 9.26 | -24.09 |
| Gwiligwili | Downstream | Border barb | 9.29 | -23.45 |
| Gwiligwili | Downstream | Border barb | 9.07 | -23.37 |
| Gwiligwili | Downstream | Border barb | 9.04 | -23.92 |
| Gwiligwili | Downstream | Chubby barb | 12.47 | -24.99 |
| Gwiligwili | Downstream | Chubby barb | 9.50 | -23.90 |
| Gwiligwili | Downstream | Chubby barb | 9.13 | -23.87 |
| Gwiligwili | Downstream | Chubby barb | 9.74 | -23.92 |
| Gwiligwili | Downstream | Ground spider | 4.19 | -25.76 |
| Gwiligwili | Downstream | Ground spider | 6.92 | -23.59 |
| Gwiligwili | Downstream | Ground spider | 7.37 | -22.42 |
| Gwiligwili | Downstream | Ground spider | 6.12 | -26.10 |
| Gwiligwili | Downstream | Ground spider | 6.76 | -23.79 |
| Gwiligwili | Downstream | Ground spider | 6.83 | -24.48 |
| Gwiligwili | Downstream | Ground spider | 7.27 | -24.26 |
| Gwiligwili | Downstream | Ground spider | 7.13 | -21.21 |
| Gwiligwili | Downstream | Ground spider | 6.37 | -23.60 |
| Gwiligwili | Downstream | Ground spider | 7.97 | -25.10 |
| Gwiligwili | Downstream | Ground spider | 5.74 | -23.76 |
| Gwiligwili | Downstream | Web Spider | 8.42 | -25.41 |
| Gwiligwili | Downstream | Web Spider | 6.22 | -23.39 |
| Gwiligwili | Downstream | Web Spider | 8.02 | -24.76 |
| Gwiligwili | Downstream | Web Spider | 7.92 | -24.02 |
| Gwiligwili | Downstream | Web Spider | 5.55 | -24.71 |
| Gwiligwili | Downstream | Web Spider | 4.25 | -24.74 |
| Gwiligwili | Downstream | Web Spider | 6.89 | -22.78 |
| Gwiligwili | Downstream | Web Spider | 6.18 | -22.16 |
| Gwiligwili | Upstream | Ground spider | 7.89 | -23.92 |
| Gwiligwili | Upstream | Ground spider | 5.65 | -25.84 |
| Gwiligwili | Upstream | Ground spider | 6.00 | -26.28 |
| Gwiligwili | Upstream | Ground spider | 6.70 | -24.93 |
| Gwiligwili | Upstream | Ground spider | 6.25 | -25.14 |
| Gwiligwili | Upstream | Ground spider | 6.20 | -24.31 |
| Gwiligwili | Upstream | Ground spider | 6.47 | -25.04 |
| Gwiligwili | Upstream | Ground spider | 6.83 | -27.21 |
| Gwiligwili | Upstream | Ground spider | 5.40 | -27.23 |
| Gwiligwili | Upstream | Ground spider | 7.13 | -25.88 |
| Gwiligwili | Upstream | Ground spider | 8.37 | -24.87 |
| Gwiligwili | Upstream | Web Spider | 7.86 | -25.15 |
| Gwiligwili | Upstream | Web Spider | 4.28 | -25.39 |
| Gwiligwili | Upstream | Web Spider | 6.27 | -24.80 |
| Gwiligwili | Upstream | Web Spider | 7.06 | -25.49 |
| Gwiligwili | Upstream | Web Spider | 5.37 | -25.93 |
| Gwiligwili | Upstream | Web Spider | 5.72 | -25.85 |
| Gwiligwili | Upstream | Web Spider | 7.28 | -25.29 |
| Gwiligwili | Upstream | Web Spider | 7.37 | -27.21 |
| Gwiligwili | Upstream | Web Spider | 7.33 | -25.06 |
| Gwiligwili | Upstream | Web Spider | 7.28 | -24.29 |
| Gwiligwili | Upstream | Web Spider | 6.92 | -25.58 |
| Gwiligwili | Upstream | Web Spider | 7.48 | -25.50 |
| Lotheni | Downstream | Brown trout | 4.23 | -21.57 |
| Lotheni | Downstream | Brown trout | 4.20 | -22.25 |
| Lotheni | Downstream | Brown trout | 4.30 | -22.08 |
| Lotheni | Downstream | Brown trout | 4.13 | -22.42 |
| Lotheni | Downstream | Brown trout | 4.31 | -21.83 |
| Lotheni | Downstream | Brown trout | 4.51 | -21.79 |
| Lotheni | Downstream | Brown trout | 4.66 | -21.92 |
| Lotheni | Downstream | Brown trout | 4.37 | -22.35 |
| Lotheni | Downstream | Ground spider | 2.57 | -19.67 |
| Lotheni | Downstream | Ground spider | 4.98 | -20.11 |
| Lotheni | Downstream | Ground spider | 4.74 | -21.45 |
| Lotheni | Downstream | Ground spider | 4.59 | -21.50 |
| Lotheni | Downstream | Ground spider | 2.84 | -24.19 |
| Lotheni | Downstream | Ground spider | 4.36 | -23.49 |
| Lotheni | Downstream | Ground spider | 4.69 | -22.77 |
| Lotheni | Downstream | Web Spider | 1.01 | -21.56 |
| Lotheni | Downstream | Web Spider | 2.05 | -22.45 |
| Lotheni | Downstream | Web Spider | 4.80 | -20.16 |
| Lotheni | Downstream | Web Spider | 4.57 | -21.21 |
| Lotheni | Downstream | Web Spider | 6.19 | -19.82 |
| Lotheni | Downstream | Web Spider | 3.28 | -21.78 |
| Lotheni | Upstream | Ground spider | 3.19 | -21.79 |
| Lotheni | Upstream | Ground spider | 3.20 | -21.74 |
| Lotheni | Upstream | Ground spider | 4.57 | -20.15 |
| Lotheni | Upstream | Ground spider | 3.56 | -19.18 |
| Lotheni | Upstream | Ground spider | 4.85 | -23.31 |
| Lotheni | Upstream | Web Spider | 3.72 | -23.40 |
| Lotheni | Upstream | Web Spider | 4.32 | -20.69 |
| Lotheni | Upstream | Web Spider | 2.10 | -23.52 |
| Lotheni | Upstream | Web Spider | 2.19 | -23.12 |
| Lotheni | Upstream | Web Spider | 4.97 | -20.25 |
| Lotheni | Upstream | Web Spider | 2.71 | -24.10 |
| Lotheni | Upstream | Web Spider | 2.47 | -22.73 |
| Lotheni | Upstream | Web Spider | 6.26 | -22.34 |
| Lotheni | Upstream | Web Spider | 3.65 | -24.11 |
| Mnyameni | Downstream | Ground spider | 7.99 | -24.28 |
| Mnyameni | Downstream | Ground spider | 6.38 | -24.24 |
| Mnyameni | Downstream | Ground spider | 6.53 | -23.59 |
| Mnyameni | Downstream | Ground spider | 6.55 | -22.68 |
| Mnyameni | Downstream | Ground spider | 6.10 | -23.36 |
| Mnyameni | Downstream | Ground spider | 5.77 | -24.94 |
| Mnyameni | Downstream | Ground spider | 6.14 | -25.35 |
| Mnyameni | Downstream | Ground spider | 6.06 | -23.52 |
| Mnyameni | Downstream | Ground spider | 8.70 | -24.70 |
| Mnyameni | Downstream | Ground spider | 5.61 | -20.47 |
| Mnyameni | Downstream | Rainbow trout | 7.82 | -21.70 |
| Mnyameni | Downstream | Rainbow trout | 8.18 | -22.58 |
| Mnyameni | Downstream | Rainbow trout | 7.99 | -20.61 |
| Mnyameni | Downstream | Rainbow trout | 7.81 | -22.49 |
| Mnyameni | Downstream | Rainbow trout | 8.16 | -22.51 |
| Mnyameni | Downstream | Rainbow trout | 7.80 | -22.74 |
| Mnyameni | Downstream | Rainbow trout | 7.75 | -22.90 |
| Mnyameni | Downstream | Rainbow trout | 7.85 | -22.84 |
| Mnyameni | Downstream | Rainbow trout | 7.76 | -22.49 |
| Mnyameni | Downstream | Rainbow trout | 7.84 | -22.68 |
| Mnyameni | Downstream | Rainbow trout | 7.97 | -22.85 |
| Mnyameni | Downstream | Rainbow trout | 7.99 | -21.03 |
| Mnyameni | Downstream | Rainbow trout | 7.73 | -22.53 |
| Mnyameni | Downstream | Rainbow trout | 7.68 | -22.45 |
| Mnyameni | Downstream | Rainbow trout | 7.68 | -22.94 |
| Mnyameni | Downstream | Rainbow trout | 8.06 | -22.54 |
| Mnyameni | Downstream | Rainbow trout | 7.92 | -22.61 |
| Mnyameni | Downstream | Rainbow trout | 7.93 | -21.50 |
| Mnyameni | Downstream | Rainbow trout | 8.90 | -20.82 |
| Mnyameni | Downstream | Rainbow trout | 8.96 | -21.61 |
| Mnyameni | Downstream | Rainbow trout | 8.65 | -20.08 |
| Mnyameni | Downstream | Rainbow trout | 8.84 | -20.86 |
| Mnyameni | Downstream | Rainbow trout | 8.68 | -21.45 |
| Mnyameni | Downstream | Rainbow trout | 9.10 | -20.75 |
| Mnyameni | Downstream | Rainbow trout | 9.04 | -21.34 |
| Mnyameni | Downstream | Rainbow trout | 9.63 | -23.28 |
| Mnyameni | Downstream | Rainbow trout | 8.66 | -21.32 |
| Mnyameni | Downstream | Rainbow trout | 9.04 | -21.30 |
| Mnyameni | Downstream | Rainbow trout | 9.09 | -21.11 |
| Mnyameni | Downstream | Rainbow trout | 8.39 | -22.14 |
| Mnyameni | Downstream | Rainbow trout | 8.60 | -21.15 |
| Mnyameni | Downstream | Rainbow trout | 9.02 | -21.00 |
| Mnyameni | Downstream | Rainbow trout | 8.71 | -21.83 |
| Mnyameni | Downstream | Rainbow trout | 9.00 | -20.98 |
| Mnyameni | Downstream | Web Spider | 5.12 | -25.99 |
| Mnyameni | Downstream | Web Spider | 7.20 | -22.65 |
| Mnyameni | Downstream | Web Spider | 5.18 | -26.26 |
| Mnyameni | Downstream | Web Spider | 7.33 | -23.39 |
| Mnyameni | Downstream | Web Spider | 7.61 | -24.58 |
| Mnyameni | Downstream | Web Spider | 8.02 | -23.72 |
| Mnyameni | Upstream | Ground spider | 6.63 | -25.30 |
| Mnyameni | Upstream | Ground spider | 4.82 | -27.05 |
| Mnyameni | Upstream | Ground spider | 5.04 | -26.91 |
| Mnyameni | Upstream | Ground spider | 8.23 | -25.10 |
| Mnyameni | Upstream | Web Spider | 5.93 | -24.89 |
| Mnyameni | Upstream | Web Spider | 6.23 | -23.38 |
| Mnyameni | Upstream | Web Spider | 6.67 | -25.41 |
| Mnyameni | Upstream | Web Spider | 4.51 | -25.12 |
| Mnyameni | Upstream | Web Spider | 6.48 | -25.64 |
| Mnyameni | Upstream | Web Spider | 5.34 | -25.56 |
| Mnyameni | Upstream | Web Spider | 6.58 | -24.48 |
| Mnyameni | Upstream | Web Spider | 5.57 | -24.23 |
| Mnyameni | Upstream | Web Spider | 6.48 | -25.25 |
| Mnyameni | Upstream | Web Spider | 5.29 | -25.86 |
| Mnyameni | Upstream | Web Spider | 5.47 | -24.04 |
| Mnyameni | Upstream | Web Spider | 8.06 | -25.09 |
| Mnyameni | Upstream | Web Spider | 5.62 | -25.61 |
| Mnyameni | Upstream | Web Spider | 5.90 | -24.71 |
| Mnyameni | Upstream | Web Spider | 5.69 | -25.73 |
| Mnyameni | Upstream | Web Spider | 8.45 | -24.80 |
| Mnyameni | Upstream | Web Spider | 5.91 | -24.33 |
| Mnyameni | Upstream | Web Spider | 5.71 | -24.75 |
| Mnyameni | Upstream | Web Spider | 6.80 | -24.49 |
| Mnyameni | Upstream | Web Spider | 8.47 | -23.75 |
| Mnyameni | Upstream | Web Spider | 6.14 | -23.55 |
| Mnyameni | Upstream | Web Spider | 7.46 | -24.03 |
| Mnyameni | Upstream | Web Spider | 5.19 | -24.59 |
| Mnyameni | Upstream | Web Spider | 7.76 | -23.62 |
| Mnyameni | Upstream | Web Spider | 4.84 | -24.01 |
| Mnyameni | Upstream | Web Spider | 5.77 | -23.92 |
| Mnyameni | Upstream | Web Spider | 8.31 | -24.21 |
| Mnyameni | Upstream | Web Spider | 8.06 | -23.59 |
| Mnyameni | Upstream | Web Spider | 6.58 | -24.15 |
| Mnyameni | Upstream | Web Spider | 5.65 | -23.50 |
| Mooi | Downstream | Brown trout | 6.08 | -18.37 |
| Mooi | Downstream | Brown trout | 5.81 | -18.31 |
| Mooi | Downstream | Brown trout | 5.55 | -18.74 |
| Mooi | Downstream | Brown trout | 5.74 | -19.48 |
| Mooi | Downstream | Brown trout | 6.31 | -19.25 |
| Mooi | Downstream | Brown trout | 5.66 | -19.04 |
| Mooi | Downstream | Brown trout | 5.80 | -18.55 |
| Mooi | Downstream | Brown trout | 5.46 | -19.65 |
| Mooi | Downstream | Brown trout | 5.71 | -18.61 |
| Mooi | Downstream | Brown trout | 6.06 | -19.52 |
| Mooi | Downstream | Brown trout | 5.56 | -19.38 |
| Mooi | Downstream | Brown trout | 5.57 | -18.83 |
| Mooi | Downstream | Brown trout | 6.01 | -19.19 |
| Mooi | Downstream | Brown trout | 6.74 | -16.69 |
| Mooi | Downstream | Ground spider | 4.67 | -19.98 |
| Mooi | Downstream | Ground spider | 5.81 | -18.89 |
| Mooi | Downstream | Ground spider | 5.00 | -19.00 |
| Mooi | Downstream | Web Spider | 7.50 | -15.67 |
| Mooi | Downstream | Web Spider | 5.01 | -19.45 |
| Mooi | Downstream | Web Spider | 6.08 | -18.80 |
| Mooi | Downstream | Web Spider | 4.81 | -19.64 |
| Mooi | Downstream | Web Spider | 5.22 | -17.31 |
| Mooi | Downstream | Web Spider | 3.77 | -17.35 |
| Mooi | Downstream | Web Spider | 2.29 | -12.90 |
| Mooi | Downstream | Web Spider | 2.06 | -12.56 |
| Mooi | Downstream | Web Spider | 4.82 | -18.19 |
| Mooi | Downstream | Web Spider | 4.91 | -22.39 |
| Mooi | Downstream | Web Spider | 5.43 | -20.91 |
| Mooi | Downstream | Web Spider | 8.54 | -25.93 |
| Mooi | Downstream | Web Spider | 2.76 | -19.89 |
| Mooi | Upstream | Ground spider | 5.10 | -19.01 |
| Mooi | Upstream | Ground spider | 4.13 | -21.97 |
| Mooi | Upstream | Ground spider | 6.11 | -17.25 |
| Mooi | Upstream | Ground spider | 5.85 | -19.68 |
| Mooi | Upstream | Web Spider | 4.81 | -21.06 |
| Mooi | Upstream | Web Spider | 5.55 | -20.07 |
| Mooi | Upstream | Web Spider | 6.58 | -17.31 |
| Mooi | Upstream | Web Spider | 4.48 | -22.32 |
| Mooi | Upstream | Web Spider | 7.47 | -20.21 |
| Mooi | Upstream | Web Spider | 5.45 | -21.06 |
| Mooi | Upstream | Web Spider | 4.82 | -20.86 |
| Mooi | Upstream | Web Spider | 3.88 | -21.67 |
| Mooi | Upstream | Web Spider | 4.02 | -21.62 |
| Mooi | Upstream | Web Spider | 4.71 | -20.47 |
| Mooi | Upstream | Web Spider | 4.58 | -21.27 |
| Mooi | Upstream | Web Spider | 4.33 | -21.56 |
| Mooi | Upstream | Web Spider | 4.44 | -21.91 |
| Mooi | Upstream | Web Spider | 4.11 | -22.82 |
| Mooi | Upstream | Web Spider | 4.20 | -21.10 |
| Mooi | Upstream | Web Spider | 4.97 | -21.69 |
| Mooi | Upstream | Web Spider | 4.04 | -21.62 |
| Mooi | Upstream | Web Spider | 4.83 | -21.24 |
| Mooi | Upstream | Web Spider | 4.92 | -20.85 |
| Mooi | Upstream | Web Spider | 7.10 | -21.59 |
| Sterkspruit | Downstream | Brown trout | 7.34 | -19.88 |
| Sterkspruit | Downstream | Brown trout | 7.03 | -20.20 |
| Sterkspruit | Downstream | Brown trout | 7.25 | -19.75 |
| Sterkspruit | Downstream | Brown trout | 7.48 | -19.57 |
| Sterkspruit | Downstream | Brown trout | 7.41 | -20.20 |
| Sterkspruit | Downstream | Brown trout | 7.22 | -19.80 |
| Sterkspruit | Downstream | Brown trout | 7.93 | -19.96 |
| Sterkspruit | Downstream | Brown trout | 8.21 | -20.40 |
| Sterkspruit | Downstream | Ground spider | 6.34 | -22.06 |
| Sterkspruit | Downstream | Ground spider | 6.59 | -22.07 |
| Sterkspruit | Downstream | Ground spider | 6.81 | -21.93 |
| Sterkspruit | Downstream | Mountain catfish | 8.90 | -20.37 |
| Sterkspruit | Downstream | Mountain catfish | 8.81 | -20.36 |
| Sterkspruit | Downstream | Mountain catfish | 8.70 | -20.35 |
| Sterkspruit | Downstream | Mountain catfish | 8.02 | -20.24 |
| Sterkspruit | Downstream | Mountain catfish | 8.55 | -20.24 |
| Sterkspruit | Downstream | Mountain catfish | 8.21 | -20.18 |
| Sterkspruit | Downstream | Mountain catfish | 9.17 | -20.18 |
| Sterkspruit | Downstream | Mountain catfish | 8.26 | -20.16 |
| Sterkspruit | Downstream | Mountain catfish | 8.87 | -20.13 |
| Sterkspruit | Downstream | Web Spider | 5.11 | -22.07 |
| Sterkspruit | Downstream | Web Spider | 5.19 | -19.93 |
| Sterkspruit | Downstream | Web Spider | 5.97 | -24.95 |
| Sterkspruit | Downstream | Web Spider | 3.13 | -14.53 |
| Sterkspruit | Downstream | Web Spider | 6.34 | -23.28 |
| Sterkspruit | Upstream | Ground spider | 2.58 | -24.13 |
| Sterkspruit | Upstream | Ground spider | 2.67 | -24.12 |
| Sterkspruit | Upstream | Ground spider | 5.51 | -20.38 |
| Sterkspruit | Upstream | Web Spider | 4.73 | -22.74 |
| Sterkspruit | Upstream | Web Spider | 4.22 | -22.23 |
| Sterkspruit | Upstream | Web Spider | 2.52 | -24.86 |
